# Construction and Functional Evaluation of a Three-Dimensional Blood–Brain Barrier Model Equipped With Human Induced Pluripotent Stem Cell-Derived Brain Microvascular Endothelial Cells

**DOI:** 10.1007/s11095-022-03249-3

**Published:** 2022-04-11

**Authors:** Toshiki Kurosawa, Daiki Sako, Yuma Tega, Yasuyuki Debori, Yumi Tomihara, Kazunobu Aoyama, Yoshiyuki Kubo, Nobuyuki Amano, Yoshiharu Deguchi

**Affiliations:** 1 Laboratory of Drug Disposition and Pharmacokinetics, Faculty of Pharma-Sciences, Teikyo University, 2-11-1 Kaga, Itabashi, Tokyo 173-8605 Japan; 2 Axcelead Drug Discovery Partners Inc., 26-1, Muraoka-Higashi 2-chome Fujisawa, Kanagawa, 251-0012 Japan

**Keywords:** 3D culture, blood–brain barrier, human iPS cells, organ-on-a-chip, transporter

## Abstract

**Purpose:**

The purpose of this study was to construct and validate an *in vitro* three-dimensional blood–brain barrier (3DBBB) model system equipped with brain microvascular endothelial cells derived from human induced pluripotent stem cells (hiPS-BMECs).

**Methods:**

The 3D-BBB system was constructed by seeding hiPS-BMECs onto the capillary lane of a MIMETAS OrganoPlate^®^ 3-lane coated with fibronectin/collagen IV. hiPS-BMECs were incubated under continuous switchback flow with an OrganoFlow^®^ for 2 days. The 3D capillary structure and expression of tight-junction proteins and transporters were confirmed by immunocytochemistry. The mRNA expression of transporters in the 3D environment was determined using qRT-PCR, and the permeability of endogenous substances and drugs was evaluated under various conditions.

**Results and Discussion:**

The expression of tight-junction proteins, including claudin-5 and ZO-1, was confirmed by immunohistochemistry. The permeability rate constant of lucifer yellow through hiPS-BMECs was undetectably low, indicating that paracellular transport is highly restricted by tight junctions in the 3D-BBB system. The mRNA expression levels of transporters and receptors in the 3D-BBB system differed from those in the 2D-culture system by 0.2- to 5.8-fold. The 3D-cultured hiPS-BMECs showed asymmetric transport of substrates of BCRP, CAT1 and LAT1 between the luminal (blood) and abluminal (brain) sides. Proton-coupled symport function of MCT1 was also confirmed.

**Conclusion:**

The 3D-BBB system constructed in this study mimics several important characteristics of the human BBB, and is expected to be a useful high-throughput evaluation tool in the development of CNS drugs.

**Supplementary Information:**

The online version contains supplementary material available at 10.1007/s11095-022-03249-3.

## Introduction

The World Health Organization (WHO) reports that more than one billion people in the world suffer from central nervous system (CNS) diseases, such as Alzheimer’s and Parkinson’s diseases, and the cost of their treatment is substantial (1–3). There are many obstacles to the development of effective therapeutic drugs for CNS diseases, and in particular, incomplete knowledge of the blood–brain barrier (BBB) properties in humans is a key issue. Many transporters and receptors are expressed at the BBB to control the exchange of drugs and nutrients between the blood and brain (4). For example, ATP-binding cassette (ABC) transporters, such as P-glycoprotein (P-gp; ABCB1) and breast cancer resistance protein (BCRP; ABCG2), prevent the entry of drugs and toxic agents into the brain interstitial fluid from the blood (5). In contrast, solute carrier (SLC) transporters such as glucose transporter 1 (GLUT1; SLC2A1), cationic amino acid transporter 1 (CAT1; SLC7A1), L-type amino acid transporter 1 (LAT1; SLC7A5), and proton/organic cation antiporter(s) regulate the exchange of nutrients, hormones, and some drugs between the blood and brain (6–10). Thus, an understanding of transporter functions at the human BBB is critical to facilitate the development of CNS drugs. However, studying the BBB in humans presents both clinical and ethical difficulties. Although many models of the BBB have been developed, including three-dimensional (3D) culture models such as spheroids (11), these do not fully reflect the three-dimensional cellular organization and direct cell–cell interactions that are critical for proper cellular differentiation and polarization, and there is still a need for a 3D model system that can mimic the complex *in vivo* microenvironment of the human BBB, where functionality is influenced by multiple factors, including capillary 3D structure and blood flow-induced shear stress (12).

Primary-cultured brain microvascular endothelial cells (BMECs) and immortalized human BMECs have been used as human BBB model cells in many laboratories worldwide, despite their disadvantages, such as difficulty of ensuring a stable supply and failure to form tight junctions (13–15). More recently, brain microvascular endothelial cells derived from human induced pluripotent stem cells (hiPS-BMECs) have been established as human BBB model cells with strong tight-junction-forming ability (16–18). Their usefulness in functional studies on the human BBB has been confirmed; for example, we previously showed that hiPS-BMECs effectively mimic the transport function of the human BBB (19–21).

Thus, cumulative evidence indicates that hiPS-BMECs would be suitable for a new *in vitro *3D-BBB system that mimics the microenvironment of the human BBB. Three types of 3D system devices have been reported (22): these are (I) a high-content system, (II) an interconnected multichip system, and (III) a high-throughput screening system. The high-content system precisely reconfigures the microenvironment of the BBB to enable angiogenesis of BMECs and direct communication with neurons, astrocytes, and pericytes (23–25). The interconnected multichip system connects various tissue chips to evaluate the pharmacokinetics of drugs in humans on a chip (26, 27). The high-throughput system allows for large-scale screening (28–30). Among these 3D systems, the high-throughput system is particularly important in the field of drug development, since many candidate compounds for CNS drugs are expected to be synthesized and screened. While fluidic 3D models with hiPS-BMECs have been reported by other groups (31, 32), our objective was to develop a hiPS-BMECs-based high-throughput system that would be suitable for preclinical studies during the development of CNS drugs. We selected the OrganoPlate^®^ 3-lane system (MIMETAS, Leiden, Netherlands) as it enables continuous-flow culture in a small-scale research environment using an OrganoFlow^®^ (MIMETAS). The most important feature of this plate is that many conditions can be evaluated in a single experiment.

Therefore, the purpose of this study was to construct an *in vitro* 3D-BBB system using a combination of hiPS-BMECs and the OrganoPlate^®^ 3-lane. To validate the constructed 3D-BBB system as a human BBB model, we also examined its tight-junction-forming ability, the mRNA expression levels of transporters and receptors known to be expressed in the human BBB, and the transport function of the transporters.

## Materials and Methods

### Reagents

Reagents used for the assay were purchased from FUJIFILM Wako Pure Chemical Industries (Osaka, Japan), Sigma-Aldrich Company (St. Louis, MO), or Tokyo Chemical Industry (Tokyo, Japan), unless otherwise specified. Arginine, L-[2, 3, 4-^3^H] ([^3^H] L-arginine, 40 Ci/mmol), and glutamic acid, L-[2, 3, 4-^3^H] ([^3^H] L-glutamate, 60 Ci/mmol) were purchased from American Radiolabeled Chemicals (St. Louis, MO). Lactic acid sodium salt, L-[^14^C(U)] ([^14^C] L-lactate, 150.6 mCi/mmol) was obtained from Perkin Elmer (Waltham, MA). JPH203 and AZD3965 were obtained from Selleck Chemicals (Houston, TX).

### Cell Culture

The human iPS cell line (IMR90-C4) was purchased from WiCell Research Institute (Madison, WI) with a material transfer agreement (MTA), and all experiments were performed only at Teikyo University and strictly managed according to the MTA. Human iPS cells were cultured on Matrigel (Corning, NY)-coated 6-well plates in mTeSR1-cGMP medium (StemCell Technologies, Vancouver, BC, Canada). The hiPS cells were maintained via daily replacement of fresh mTeSR1-cGMP and passaged with 0.5 mM EDTA in PBS. The differentiation of hiPS cells into hiPS-BMECs was performed as previously reported (16, 17). Briefly, hiPS cells were dissociated with Accutase (Merck-Millipore, Billerica, MA) and seeded at a density of 1.0 – 1.2 × 10^5^ cells/well on Matrigel-coated 6-well plates (Thermo Fisher Scientific, Waltham, MA) in mTeSR1-cGMP containing 10 μM Y27632 (FUJIFILM Wako), a ROCK inhibitor (day -3). At 24 h after seeding, the medium was replaced with fresh mTeSR1-cGMP without Y27632 (day − 2). On day 0, the medium was switched from mTeSR1-cGMP to unconditioned medium (UM; see Supplementary Material for details of the components) and changed daily until day 5. From day 6, the medium was changed from UM to human endothelial serum-free medium (ESFM; Thermo Fisher Scientific) containing 1% human serum from platelet-poor human plasma (hPDS; Sigma-Aldrich Company), 10 μM all-trans-retinoic acid (RA; FUJIFILM Wako), and 20 ng/mL human fibroblast growth factor 2 (FGF2; Sigma-Aldrich Company) until day 8. On day 8, the cells were dissociated via Accutase treatment and seeded onto OrganoPlate^®^ 3-lane plates. The detailed protocol for the construction of the 3D-BBB system is described in the next section and Supplementary Material. On day 9, the medium was replaced with fresh ESFM + 1% hPDS. On day 10, various experiments were performed using the 3D-BBB system. The cells were maintained at 37°C in an atmosphere of 95% air and 5% CO_2_. The 2D culture was constructed by seeding hiPS-BMECs on Transwells (Cell Culture Insert, Corning) coated with fibronectin/collagen IV solution (both 0.1 mg/mL, PharmaCo-Cell, Nagasaki, Japan) according to the reported method (19). The experimental apparatus and reagents used in this study are summarized in the Supplementary Material.

### Construction of the 3D-BBB System

The 3D-BBB system using the OrganoPlate^®^ 3-lane plate was constructed with some modifications to the protocol published by MIMETAS. During the differentiation of hiPS-BMECs, to separate the capillary side from the brain side, extracellular matrix hydrogel composed of 4 mg/mL collagen I (Cultrex 3D Matrix Rat Collagen-I, 5 mg/mL, AMSbio), 100 mM HEPES, and 3.7 mg/mL NaHCO_3_ was dispensed into the middle lane. The plate was incubated at 37°C for 15 min to polymerize collagen I. After incubation, HBSS (+) without phenol red (Fujifilm Wako) was added to the gel channel to prevent the gel from drying out. On day 8, a suspension of hiPS-BMECs at a density of 1.0 × 10^7^ cells/mL was prepared with ESFM + 1% hPDS, RA, and FGF2 containing 10 μM Y27632 and 10 μM GM6001 (Abcam, Cambridge, MA), a broad spectrum MMP inhibitor, to improve cell adhesion and prevent cell invasion into the gel, respectively. The suspension was injected into capillary lanes precoated with fibronectin/collagen IV solution (PharmCo-Cell). After seeding the cells, the plate was incubated for 4 h at a tilt to allow the cells to adhere to the gel. After incubation, the medium was added to the capillary channels and the plate was placed on an OrganoFlow^®^ (MIMETAS), switching between a + 7° and − 7° inclination every 8 min to create a bidirectional flow mimicking blood flow. On day 9, the medium in the capillary channels was replaced with fresh ESFM + 1% hPDS containing 10 μM MMP inhibitor. The plate was placed on an OrganoFlow^®^ from days 8 to 10 for incubation under continuous flow to be as comparable as possible with the Transwell model, and the difference between 2D culture with Transwells and 3D culture with OrganoPlate^®^ 3-lane was caused by the presence or absence of flow between day 8 and day 10.

### Quantitative Reverse Transcription-Polymerase Chain Reaction (qRT-PCR)

RNA was isolated from 2D- and 3D-cultured hiPS-BMECs on day 10 and cDNA was synthesized using the SYBR Green Fast Advanced Cell-to-CT Kit (Thermo Fisher Scientific) according to the manufacturer’s instructions. The lysis solution contained 1:100 DNase I to fully digest genomic DNA. qPCR reaction was performed in a volume of 10 μL with 400 nM of each primer, 2 μL cDNA, 5 μL PowerUp SYBR Green Master Mix (Thermo Fisher Scientific) on an Applied Biosystems 7500 Real-Time PCR System (Thermo Fisher Scientific) with the following thermocycling conditions; 1 cycle of UDG activation at 50°C for 2 min, 1 cycle of enzyme activation at 95°C for 10 min, and 40 cycles of PCR reaction [95°C for 3 s, 60°C for 30 s]. In 3D-cultured hiPS-BMECs, cells were collected from 20 to 30 chips per plate. Primer sequences are given in the Supplementary Material. Relative mRNA expression levels were calculated by the ΔCt method based on the mRNA level of glyceraldehyde 3-phosphate dehydrogenase (GAPDH), a housekeeping gene. Amplification and detection were performed using an Applied Biosystems 7500 Real-Time PCR System (Thermo Fisher Scientific). Targets for which PCR amplification products were not obtained and Ct values could not be calculated were designated as “under the limit of quantification (ULQ)”.

### Permeability Experiments

Permeability experiments were performed on day 10, at 48 h after seeding hiPS-BMECs on the plate. After removal of the medium in the capillary channels, the assay buffer (pH 7.4) was added to both sides of the capillary and brain channels and preincubated on the OrganoFlow^®^ for 30 min. The assay buffer containing test drugs was added to the donor side to initiate the assay, and the plate was then incubated on the OrganoFlow^®^ at 37°C for the designated times. Transporter inhibitors were added to both the donor and receiver sides at the start of preincubation. In all permeability experiments, the amount of lucifer yellow transported through the paracellular pathway to the receiver side was measured with a SpectraMax i3x (Molecular Devices, San Jose, CA) as a measure of cell-to-cell tightness. The protocol for the permeability experiments, including the composition of the assay buffer, is described in detail in the Supplementary Material. The apparent permeability coefficient (*P*_app_) was calculated as follows:

$${P}_{app}= (dQ / dt) / D_{0} \times S$$

where d*Q*/d*t*, *D*_0_, and S are the transport velocity of the test drugs, the initial concentration of test drugs on the donor side, and the surface area of the extracellular matrix hydrogel in the middle lane, respectively. The efflux ratio value was calculated from the *P*_app_ value in the capillary-to-brain (A-to-B) and brain-to-capillary (B-to-A) directions as follows:

$$Efflux\ ratio = {P}_{app,\ B-to-A}/ {P}_{app,\ A-to-B}.$$

### Immunocytochemistry

After removal of the medium, the capillary lane was washed with PBS into the capillary channels. hiPS-BMECs were fixed with 4% paraformaldehyde (PFA)/PBS for 10 min, washed with PBS, and permeabilized with 0.1% saponin/PBS for 15 min on the OrganoFlow^®^ at room temperature, followed by overnight incubation at 4°C with the following primary antibodies: anti-VE-cadherin (D87F2, diluted 1/50; Cell Signaling Technology, Danvers, MA), anti-GLUT1 (SPM498, diluted 1/100; Abcam), anti-claudin-5 (4C3C2, diluted 1/100; Thermo Fisher Scientific), anti-ZO-1 (diluted 1/50; Thermo Fisher Scientific), anti-BCRP (5D3, diluted 1/200; Merck-Millipore), and anti-LAT1 (diluted 1/50; Atlas Antibodies, Stockholm, Sweden). The capillary and brain lanes were washed with PBS on the OrganoFlow^®^, and the hiPS-BMECs were incubated with DAPI (diluted 1/1000) and a secondary antibody labeled with Alexa Fluor 488 (diluted 1/600; Thermo Fisher Scientific) or Cy3 red (diluted 1/600; Jackson ImmunoResearch, West Grove, PA) at room temperature for 1 h. After washing with PBS, fluorescently labeled cells were encapsulated in PermaFluor Aqueous Mounting Medium (Thermo Fisher Scientific). For staining with CellMask™ Green Plasma Membrane Stain (Thermo Fisher Scientific), hiPS-BMECs were fixed with 4% PFA/PBS after reaction with the reagents according to the manufacturer’s instructions. All fluorescence images were captured with a confocal imaging cytometer CQ1 (Yokogawa Electric Corporation, Tokyo, Japan) using 10 × objectives. Images were constructed using CQ1 and ImageJ software. FITC for CellMask, Alexa Fluor, and Cy3 were excited by 405, 488 and 561 nm lasers, and emission was selected with BP447/60, BP525/50 and BP617/73 filters, respectively.

### Quantification of Test Drugs

The test drugs were quantified using a high-performance liquid chromatography (HPLC)-tandem mass spectrometry system composed of a Nexera-XR (Shimazu, Kyoto, Japan) HPLC system connected to a Qtrap4500 (AB Sciex, Foster City, CA, USA) mass spectrometer with an electrospray ionization interface. The conditions are described in detail in the Supplementary Material. ^3^H and ^14^C-labeled samples were prepared as scintillation mixtures with Hionic-Fluor (Perkin Elmer) and the radioactivity was counted in a liquid scintillation counter (Tri-Carb 3110TR, Perkin Elmer).

### Statistical Analysis

The statistical significance of differences was determined using an unpaired two-tailed Student’s t-test. Values of *P* < 0.05, 0.01, and 0.001 were considered to represent statistically significant differences. All values are presented as the mean ± standard error.

## Results

### Construction of 3D-BBB System Using hiPS-BMECs

**Fig. 1:**
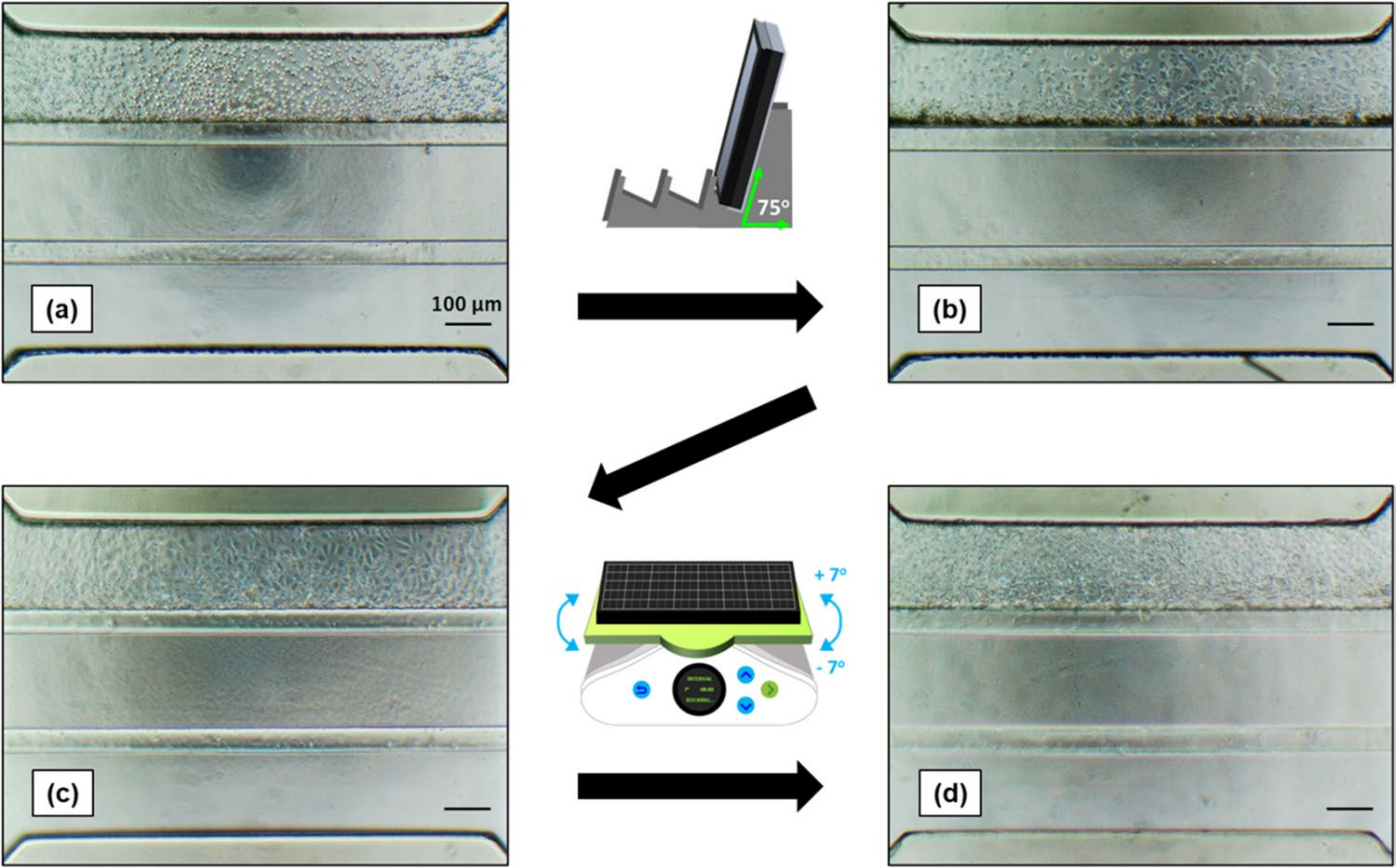
Scheme for the construction of the three-dimensional blood–brain barrier (3D-BBB) system with the OrganoPlate^®^ 3-lane. (**a**) On day 8 of the differentiation period of brain microvascular endothelial cells derived from human induced pluripotent stem cells (hiPS-BMECs), cells were seeded into the capillary lane. (**b**) The plate was tilted and incubated at 37°C for 4 h to allow the hiPS-BMECs to adhere to the gel in the middle lane. When the hiPS-BMECs were attached to the gel, flow culture was started on the OrganoFlow^®^. (**c**, **d**) Images of hiPS-BMECs after 24 h (day 9) and 48 h (day 10) of flow culture. The scale bar is 100 μm.

**Fig. 2:**
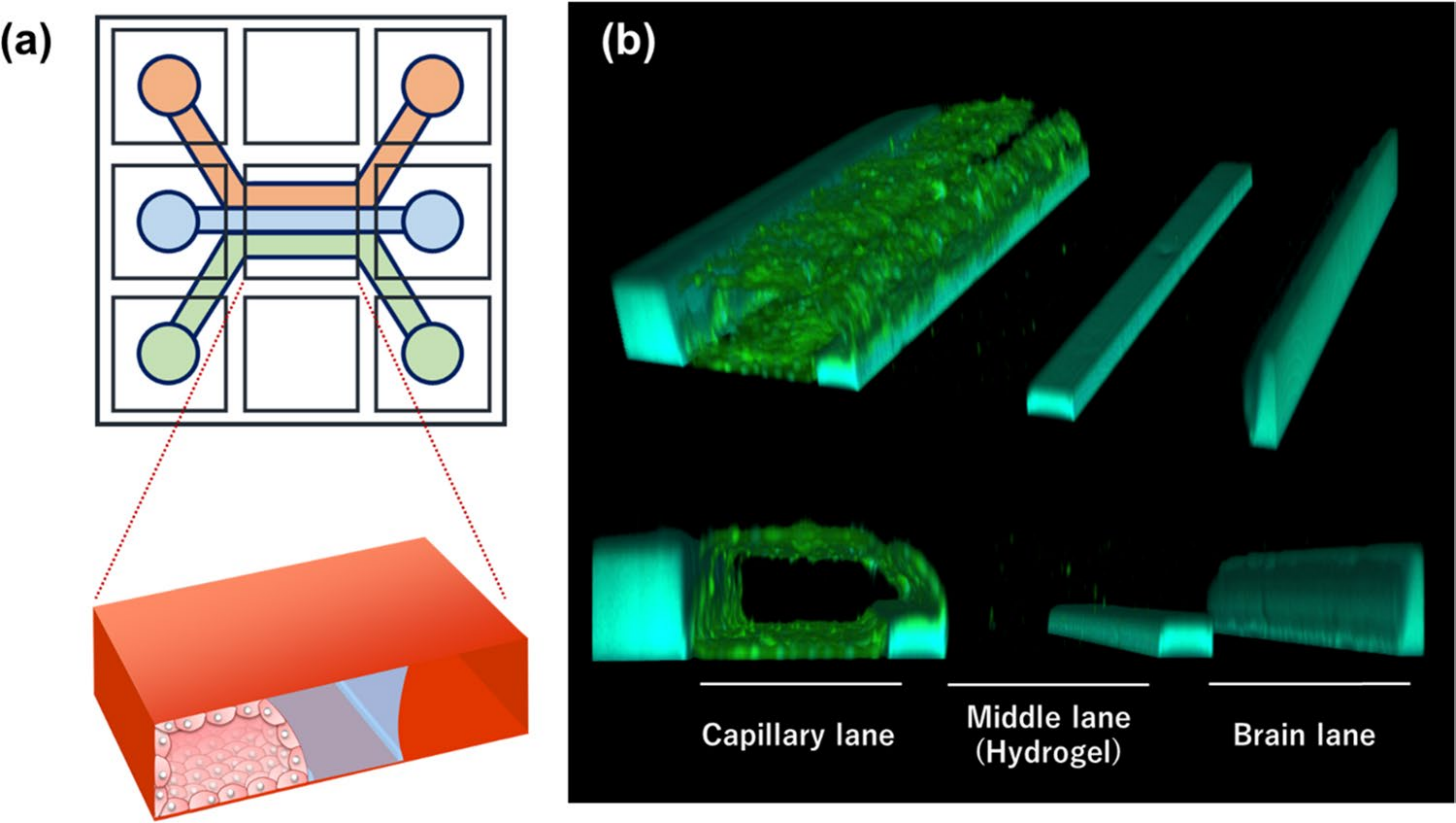
Overall view of OrganoPlate^®^ 3-lane. (**a**) Illustration of the OrganoPlate^®^ 3-lane. The plate consists of 40 clusters of 9 wells, each divided into three microfluidic lanes. There are 40 of these nine-well clusters in one plate, allowing for high-throughput studies. Red lane, capillary lane; blue lane, middle (hydrogel) lane; green lane, brain lane. (**b**) Cell membrane staining of hiPS-BMECs seeded on the OrganoPlate^®^ 3-lane. Green, cell membrane via CellMask staining; light blue, polymers that make up the walls and guides of the plate.

**Fig. 3:**
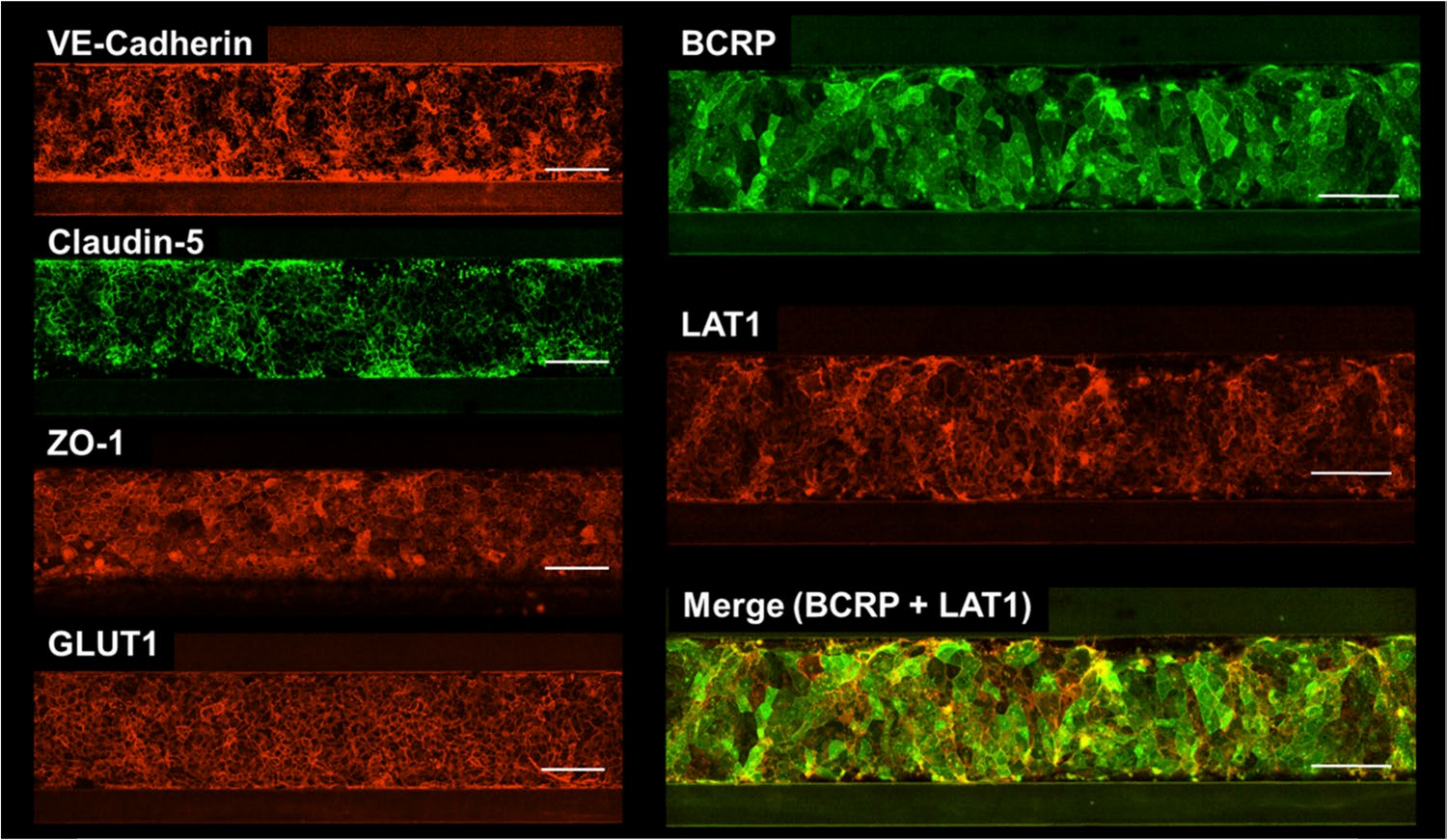
Immunocytochemistry of brain microvascular endothelial cells derived from human induced pluripotent stem cells (hiPS-BMECs) seeded on the three-dimensional blood–brain barrier (3D-BBB) system. Immunostaining for VE-cadherin, claudin-5, ZO-1, BCRP, LAT1, and GLUT1 in hiPS-BMECs seeded in the OrganoPlate^®^ 3-lane. The stained image was taken at the bottom of the capillary lane. The scale bar is 100 μm.

To construct the 3D-BBB system with hiPS-BMECs, the capillary lane of an OrganoPlate^®^ 3-lane plate was first coated with fibronectin/collagen IV. The hiPS-BMECs, which were attached to the hydrogel surface by tilting the plate, formed capillary structures in continuous flow culture (Fig. 1). The OrganoPlate^®^ 3-lane was divided into nine wells, and the center well was captured (Fig. 2a). The capillary structure was confirmed by staining with CellMask (Fig. 2b). Immunocytochemistry showed that VE-cadherin (an endothelial cell marker), claudin-5 and ZO-1 (tight junction proteins), and the transporters GLUT1, BCRP, and LAT1 were expressed in hiPS-BMECs on 3D-BBB system plates (Fig. 3).

### Formation of Tight Junctions in 3D-BBB System with hiPS-BMECs

**Fig. 4:**
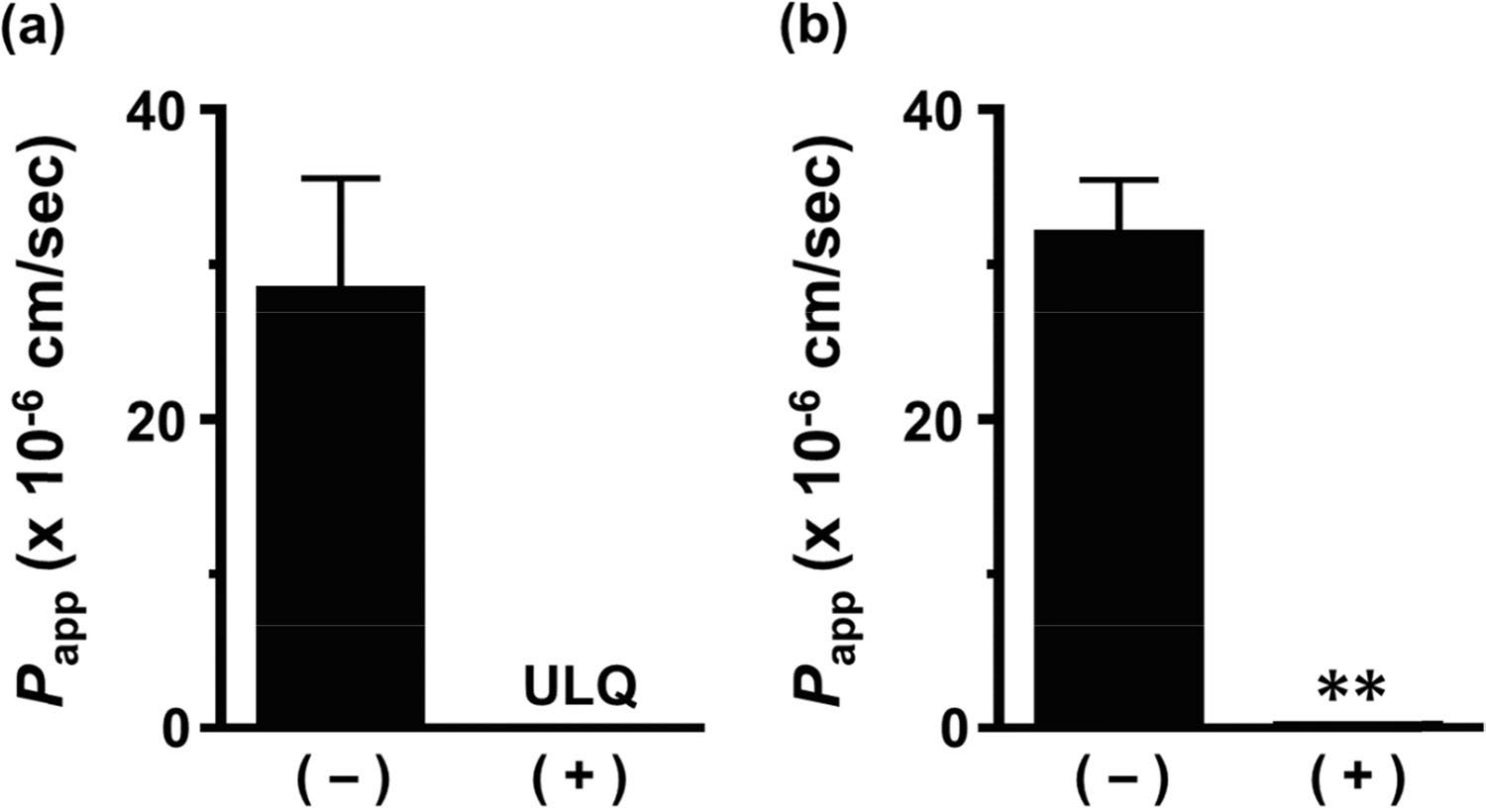
Apparent permeability coefficient (*P*_app_) of lucifer yellow in brain microvascular endothelial cells derived from human induced pluripotent stem cells (hiPS-BMECs) seeded on the three-dimensional blood–brain barrier (3D-BBB) system. The permeability of lucifer yellow in the A-to-B direction was measured at 5 h (**a**) and 7 h (**b**). The ( +) and (-) columns show the *P*_app_ of lucifer yellow in the presence and absence of hiPS-BMECs, respectively. Each value represents the mean ± standard error of 7–9 experiments. ^**^P < 0.01 indicates a significant difference *versus* the *P*_app_ of lucifer yellow without hiPS-BMECs. ULQ, under the limit of quantification.

The ability of the 3D-BBB system to form tight junctions was evaluated by measuring the transport of lucifer yellow, a paracellular marker. The transport of lucifer yellow in the A-to-B direction was undetectable up to 5 h, and the *P*_app_ was 0.189 × 10^−6^ cm/s at 7 h (Fig. 4). In contrast, the *P*_app_ of lucifer yellow in the hydrogel without hiPS-BMECs was 28.3 × 10^–6^ cm/s at 5 h and 31.9 × 10^–6^ cm/s at 7 h.

### Relative mRNA Expression Levels of Transporters and Receptors in hiPS-BMECs on 3D-BBB System

The relative mRNA expression levels of transporters and receptors were quantified by qRT-PCR in 3D (3D-BBB system)- and 2D (Transwell)-cultured hiPS-BMECs, and the results are summarized in Table I. The mRNA expression levels in the 3D-cultured hiPS-BMECs were in the range of 0.2 to 5.8 times those in the 2D-cultured hiPS-BMECs, indicating that 3D-culture has a relatively minor influence on expression of these mRNAs. Among the genes encoding nutrient transporters, GLAST, GLUT3, LAT1, and MCT1 were highly expressed in both 3D-cultured and 2D-cultured hiPS-BMECs. The mRNA expression ratio (3D/2D) of CAT1 was 3.8, and its expression in the 3D culture was significantly higher than that in the 2D culture. Among ABC transporters, P-gp was hardly expressed, whereas BCRP and MRP5 tended to be more highly expressed in 3D-cultured hiPS-BMECs than in 2D-cultured hiPS-BMECs. Most organic anion/cation transporters were undetectable, except for OATP2A1 and OCTN2, which showed relatively high expression. Among the receptors, LRP1 and GLP1R showed relatively high mRNA expression.

**Table I:** Relative mRNA Expression Levels of Transporters and Receptors in 3D-Cultured and 2D-Cultured hiPS-BMECs

Gene (Protein name)	mRNA expression (Target mRNA/GAPDH mRNA)						Expression Ratio (3D/2D)
	3D culture			2D culture			
ABCB1 (P-gp)	0.0000146	±	0.00000365	0.00000475	±	0.00000171	3.09
ABCC1 (MRP1)	ULQ			ULQ			-
ABCC4 (MRP4)	0.00381	±	0.00123	0.00272	±	0.00106	1.40
ABCC5 (MRP5)	0.0115	±	0.0009	0.00198	±	0.00074	5.82^*^
ABCG2 (BCRP)	0.115	±	0.032	0.0350	±	0.0120	3.28^*^
SLC1A3 (GLAST)	0.256	±	0.087	0.298	±	0.058	0.859
SLC2A1 (GLUT1)	0.0677	±	0.0117	0.0803	±	0.0203	0.843
SLC2A3 (GLUT3)	0.179	±	0.067	0.0872	±	0.0322	2.05
SLC3A2 (4F2hc)	0.0721	±	0.0070	0.0417	±	0.0093	1.73
SLC5A7 (CHT1)	0.0407	±	0.0211	0.0266	±	0.0040	1.53
SLC7A1 (CAT1)	0.0205	±	0.0014	0.00534	±	0.00143	3.83^*^
SLC7A5 (LAT1)	0.263	±	0.030	0.190	±	0.021	1.38
SLC7A6 (LAT2)	ULQ			ULQ			-
SLC16A1 (MCT1)	0.135	±	0.046	0.145	±	0.049	0.928
SLC16A7 (MCT2)	0.000751	±	00,000,259	ULQ			-
SLC19A1 (RFC1)	0.0119	±	0.0028	0.0413	±	0.0121	0.288^*^
SLCO1A2 (OATP1A2)	ULQ			ULQ			-
SLCO2A1 (OATP2A1)	0.0234	±	0.0089	0.0531	±	0.0204	0.440
SLCO1B1 (OATP1B1)	ULQ			ULQ			-
SLC22A1 (OCT1)	0.00188	±	0.00045	0.00682	±	0.00179	0.276^*^
SLC22A2 (OCT2)	ULQ			ULQ			-
SLC22A3 (OCT3)	0.00164	±	0.00107	0.00246	±	0.00068	0.664
SLC22A4 (OCTN1)	0.000169	±	0.000075	0.000205	±	0.000113	0.826
SLC22A5 (OCTN2)	0.0114	±	0.0035	0.0444	±	0.0163	0.257
SLC22A6 (OAT1)	ULQ			ULQ			-
SLC22A7 (OAT2)	0.00596	±	0.00260	0.00291	±	0.00134	2.05
SLC22A8 (OAT3)	ULQ			ULQ			-
SLC29A1 (ENT1)	0.0318	±	0.0114	0.0911	±	0.0378	0.349
SLC29A2 (ENT2)	0.0161	±	0.0052	0.0215	±	0.0055	0.751
SLC29A4 (PMAT)	0.00833	±	0.00066	0.0150	±	0.0031	0.555
SLC35F2	0.00313	±	0.00004	0.00793	±	0.00178	0.395
SLC43A3 (ENBT1)	0.00305	±	0.00057	0.00853	±	0.00055	0.358^*^
SLC44A1 (CTL1)	0.0483	±	0.0078	0.129	±	0.044	0.374
SLC44A2 (CTL2)	0.0941	±	0.0066	0.213	±	0.041	0.442^*^
SLC47A1 (MATE1)	ULQ			ULQ			-
SLC47A2 (MATE2)	0.0211	±	0.0126	0.00498	±	0.00085	4.24
OSCP1	0.00249	±	0.00097	0.0125	±	0.0045	0.199
LEPR	ULQ			ULQ			-
LRP1	0.158	±	0.071	0.276	±	0.124	0.573
TFRC	ULQ			ULQ			-
RAGE	0.00536	±	0.00181	0.00287	±	0.00145	1.86
INSR	0.00902	±	0.00248	0.0161	±	0.0003	0.561^*^
GLP1R	0.245	±	0.110	0.0496	±	0.0099	4.95

mRNA expression levels in 3D- and 2D-cultured hiPS-BMECs were normalized by the value of GAPDH. Each value represents the mean ± SE of 3–11 experiments. ^*^P < 0.05 indicate a significant difference in mRNA expression levels between 3 and 2D cultures. ULQ; under the limit of quantification

### Evaluation of Permeability by Passive Diffusion in 3D-BBB System

The passive diffusion marker antipyrine showed similar levels of transport in the A-to-B and B-to-A directions up to 4 h. The *P*_app_ values of antipyrine were 33.4 × 10^−6^ cm/s and 35.1 × 10^−6^ cm/s in the A-to-B and B-to-A directions, respectively, suggesting similar permeability in both directions (Table II).

**Table II:** The *P*_app_ Values of Drugs and Endogenous Substances in the 3D-BBB System

Drug	Condition	*P*_app_ (× 10^–6^ cm/s)						Efflux ratio (B-to-A/A-to-B)
		A-to-B			B-to-A			
Quinidine (1 μM)	Control	21.6	±	1.5	22.8	±	0.9	1.06
Atenolol (30 μM)	Control	0.822	±	0.297	0.633	±	0.107	0.771
Dantrolene (2 μM)	Control	14.1	±	1.3	50.9	±	6.5^***^	3.60
Cladribine (10 μM)	Control	2.69	±	0.30	15.1	±	2.9^**^	5.61
	+ 1 μM Ko143	5.32	±	0.96	7.70	±	1.23	1.45
[^3^H] L-Arginine (3 μCi/mL)	Control	14.1	±	1.9	5.77	±	0.70	0.410
[^3^H] L-Glutamate (3 μCi/mL)	Control	6.21	±	0.59	6.10	±	0.35	0.982
[^14^C] L-Lactate (1 μCi/mL)	A: pH 7.4—B: pH 7.4	19.1	±	2.2	18.1	±	1.5	0.944
	A: pH 6.0—B: pH 7.4	34.1	±	0.8	8.74	±	0.52^***^	0.257
	+ 20 nM AZD3965	12.0	±	0.5^†††^				
	A: pH 7.4—B: pH 6.0	7.68	±	1.92	23.6	±	0.8^**^	3.07
	+ 20 nM AZD3965				9.60	±	0.39^††^	
Gabapentin (10 μM)	Control	16.0	±	1.7	6.40	±	0.40^**^	0.401
	+ 10 μM JPH203	1.45	±	0.31	2.68	±	0.49	1.85
Antipyrine (2 μM)	Control	33.4	±	1.0	35.1	±	4.9	1.05

Inhibitors were added to both capillary and brain lanes. Each value represents the mean ± standard error of four to eight experiments. ^**^P < 0.01 and ^***^P < 0.001 indicate a significant difference *versus* *P*_app_ in the A-to-B direction. ^††^*P* < 0.01 and ^†††^*P* < 0.001 indicated a significant difference *versus* *P*_app_ in the A-to-B or B-to-A direction under the same conditions

### Evaluation of ABC Transporter-Mediated Transport in 3D-BBB System

**Fig. 5:**
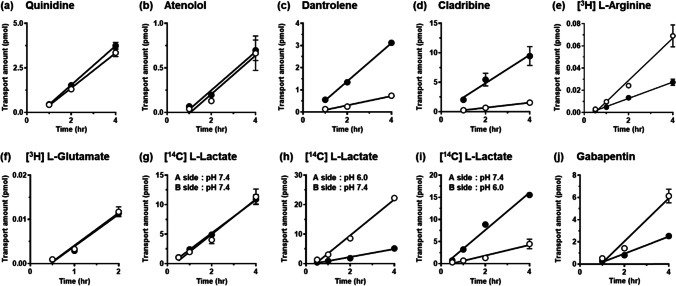
Permeability of drugs and endogenous nutrients in three-dimensional blood–brain barrier (3D-BBB) system under same conditions without inhibitor. Transcellular transport of drugs and endogenous nutrients in the capillary lane (A-side) and brain lane (B-side) across brain microvascular endothelial cells derived from human induced pluripotent stem cells (hiPS-BMECs). The directional transport of quinidine, atenolol (**a**, **b**; P-gp substrate), dantrolene, cladribine (**c**, **d**; BCRP substrate), [^3^H] L-arginine (**e**; CAT1 substrate), [^3^H] L-glutamate (**f**; GLAST substrate), [^14^C] L-lactate (**g-i**; MCT1 substrate), and gabapentin (**j**; LAT1 substrate) was measured for up to 2 or 4 h. Open and closed circles show transport in the A-to-B (capillary lane-to-brain lane) and B-to-A (brain lane-to-capillary lane) directions, respectively. The transport of [^14^C] L-lactate was performed at pH 7.4, for both the capillary and brain lanes (**g**), at pH 6.0 for the capillary lane (**h**), and at pH 6.0 for the brain lane (**i**). Each value represents the mean ± standard error of 4–8 experiments.

The transported amounts of quinidine and atenolol, substrates of P-gp, were comparable in the A-to-B and B-to-A directions with efflux ratio values (B-to-A/A-to-B) of 1.06 and 0.771, respectively (Fig. 5a, b and Table II). The transported amounts of dantrolene and cladribine, substrates of BCRP, showed asymmetric transport with significantly greater transport in the B-to-A direction than in the A-to-B direction; their efflux ratios were calculated to be 3.60 and 5.61, respectively (Fig. 5c, d and Table II). The efflux ratio of cladribine was reduced to 1.45 in the presence of the BCRP inhibitor Ko143 (Table II).

### Evaluation of SLC Transporter-Mediated Transport in 3D-BBB System

The transported amount of [^3^H] L-arginine, a substrate of CAT1, in the A-to-B direction was greater than that in the B-to-A direction, and the *P*_app_ values were calculated to be 14.1 × 10^−6^ cm/s and 5.77 × 10^−6^ cm/s, respectively (Fig. 5e and Table II). The *P*_app_ values of [^3^H] L-glutamate, a substrate of GLAST, were 6.21 × 10^−6^ cm/s and 6.10 × 10^−6^ cm/s, and the permeability was equal in the A-to-B and B-to-A directions (Fig. 5f). The transport of [^14^C] L-lactate, a substrate of MCT1, in each direction showed little difference when the capillary and brain side were both at pH 7.4 (Fig. 5g). However, when one lane was set to pH 6.0 (with the opposite lane set to pH 7.4; Fig. 5h, i), the *P*_app_ values of [^14^C] L-lactate in the A-to-B and B-to-A directions were calculated as 34.1 × 10^−6^ cm/s and 8.74 × 10^−6^ cm/s (capillary lane: pH 6.0 – brain lane: pH 7.4), and 7.68 × 10^−6^ cm/s and 23.6 × 10^−6^ cm/s (capillary lane: pH 7.4 – brain lane: pH 6.0), respectively. The transport promoted by the H^+^-gradient was significantly reduced by approximately 60% in the presence of AZD3965, an inhibitor of MCTs (Table II). The transport amount of gabapentin, a substrate of LAT1, in the A-to-B direction was larger than that in the B-to-A direction, with *P*_app_ values of 16.0 × 10^−6^ cm/s and 6.40 × 10^−6^ cm/s, respectively (Fig. 5j and Table II). Furthermore, the transport of gabapentin was reduced by approximately 90% and 60% in the A-to-B and B-to-A directions, respectively, in the presence of JPH203, an inhibitor of LAT1 (Table II).F

## Discussion

An *in vitro* BBB system that effectively mimics the anatomical and physiological functions of the human BBB is desirable for promoting the development of drugs to treat CNS diseases. In the present study, we constructed an innovative 3D-BBB system with a 3D culture of hiPS-BMECs to reproduce the human BBB, and our selection of the MIMETAS OrganoPlate^®^ 3-lane as the experimental device enabled us to successfully construct a 3D-BBB system suitable for high-throughput evaluation even in a small-scale environment. In recent years, various platforms based on the MIMETAS plate have become available, including a pathological model for the BBB (33).

The OrganoPlate^®^ 3-lane has three divided lanes, namely capillary, middle (hydrogel), and brain lanes, with a cluster of nine wells, as shown in Fig. 2a. Forty clusters were located on a plate. According to MIMETAS (https://www.mimetas.com/en/organoplate-3-lane-40/) and the related paper (29), this plate experiences the average shear stress of ~ 1.2 dyne/cm^2^ (0–5 dyne/cm^2^). In the early stage of the study, we found that the adhesion of hiPS-BMECs was markedly improved by coating the capillary lane with fibronectin/collagen IV and strengthening the hydrogel; optimal adhesion of hiPS-BMECs was not obtained when the protocol presented by MIMETAS (https://www.mimetas.com/en/organoplate-3-lane-40/) was used. In addition, addition of Rock inhibitor and MMP inhibitor to the culture medium improved cell adhesion and inhibited cell incursion into the gel, respectively, suggesting that these modifications successfully improved attachment of hiPS-BMECs to the capillary lane with the appropriate capillary structure under flow conditions (Fig. 1).

Cell membrane staining of the 3D-BBB system using CellMask confirmed the capillary structure (Fig. 2b), and cell-to-cell tightness was evaluated in terms of the paracellular transport of lucifer yellow. Lucifer yellow showed no leakage from the capillary lane to the brain lane up to 5 h (Fig. 4a). At 7 h, the *P*_app_ values of lucifer yellow in the presence and absence of cells were 0.189 × 10^−6^ cm/s and 31.9 × 10^−6^ cm/s, respectively, suggesting that hiPS-BMECs block the leakage of lucifer yellow by more than 99% (Fig. 4b). In 2D culture of hiPS-BMECs, the *P*_app_ of lucifer yellow was 0.0845 × 10^–6^ cm/s, and the transendothelial electrical resistance (TEER) value was over 2000 Ω·cm^2^ (19), suggesting that the ability to form tight junctions is maintained in the 3D-BBB system. This is comparable to that in a previous report on a 3D culture model of human BBB cells (34, 35). Furthermore, immunochemistry confirmed protein expression of claudin-5 and ZO-1, which are tight-junction components, in hiPS-BMECs on the 3D-BBB system (Fig. 3). These results clearly indicate that the constructed experimental system has the ability to form strong tight junctions, which is essential for high-throughput evaluation.

In the permeability experiment with antipyrine, a passive diffusion marker, permeability in the A-to-B and B-to-A directions showed no significant difference, and this suggests that construction of the 3D-BBB system had little effect on drug permeability (Table II). However, it remains important to precisely clarify transporter expression and transport function in the constructed 3D-BBB system, since it has been reported that BBB function is affected by the three-dimensional capillary structure and the shear stress caused by blood flow. In addition, it has been reported that endothelial cells cultured in two dimensions exhibit loss of a BBB-like phenotype owing to rapid dedifferentiation (36, 37), and that shear stress changes the expression of transporters and the strength of tight junctions (12) in endothelial cells, suggesting that precise construction of a capillary structure and the creation of flow mimicking blood flow are essential to reproduce the features of the human BBB. In the present study, antipyrine was used as a passive diffusion marker, and its *P*_app_ in hydrogel was calculated to be 59.2 × 10^–6^ cm/s (data not shown), suggesting the difficulty to evaluate drugs of which permeability is greater than this upper limit (59.2 × 10^–6^ cm/s). For this, in this study, the *P*_app_ was calculated as the value including the permeability in the hydrogel to make it possible to compare with the limit. Therefore, it is suggested that the transport of drugs, with lower permeability than the limit (that of antipyrine in hydrogel), exhibits the function of the target transporter, and this is also supported by results obtained in the inhibition study of transports. In addition, the *P*_app_ in the B-to-A direction for dantrolene, a substrate of BCRP, is 50.9 × 10^–6^ cm/s, was shown to be close to the limit, and this implies the possible underestimation of its efflux ratio. The drug permeability of the hydrogel alone was not evaluated, and this may be a limitation on interpreting the permeability data in the 3D plates used in this study.

In the permeability study on hiPS-BMECs cultured in the OrganoPlate^®^ 3-lane, the transport properties of quinidine and atenolol, substrates of P-gp, were comparable in the A-to-B and B-to-A directions, with efflux ratio values of 1.06 and 0.771, respectively (Fig. 5a, b and Table II). These results suggest that P-gp (ABCB1) in hiPS-BMECs is only marginally functional even in 3D culture, since the values for quinidine in a monkey *in vitro* BBB model and the prediction for human BBB were reported to be approximately 1.7 and 13.4, respectively (38, 39). In addition, lack of P-gp function has been reported in BMECs derived from other hiPS cell lines (40). In addition to P-gp, TFRC and MRP1 mediate drug transport, and further technical advances will be needed to prepare hiPS-BMECs that adequately express them.

The mRNA expression of BCRP (ABCG2) was 3.3-fold higher in 3D-cultured hiPS-BMECs than in 2D cultures (Table I), and BCRP protein expression was also detected in the 3D-BBB system (Fig. 3). When the transport of dantrolene and cladribine, substrates of BCRP, was examined in the 3D-BBB system, the significant efflux transport from the brain seen *in vivo* was reproduced, with efflux ratios of 3.6 and 5.6, respectively (Fig. 5c, d and Table II). In particular, a previous study in rats reported an efflux ratio of 4.2 for dantrolene and also found comparable expression of BCRP in human, rat, and mouse BBBs (41, 42), supporting the results obtained in the present study. Furthermore, the efflux ratio value of cladribine was decreased to 1.45 in the presence of Ko143, a BCRP inhibitor (Table II), and the inhibition of BCRP enhanced the transport of cladribine in the A-to-B direction and lowered that in the B-to-A direction, indicating luminal localization of BCRP in hiPS-BMECs. These results suggest that the 3D-BBB system can evaluate the function of the BCRP as well as the 2D model, and may therefore prove useful in applications such as disease modeling and toxicity evaluation in the future. This is important, because BCRP is more highly expressed than P-gp in the human BBB (43), and functional changes of BCRP at the BBB are associated with various diseases (44–46).

In addition to ABC transporters, including P-gp and BCRP, SLC transporters play important roles in drug disposition (6). In the BBB, the transport of L-arginine and L-glutamate is known to occur via CAT1 (SLC7A1) and GLAST (EAAT1/SLC1A3), respectively (47). The study of mRNA expression showed 3.8-fold higher expression of CAT1 in 3D culture, and the transport of [^3^H] L-arginine was greater in the A-to-B direction than in the B-to-A direction (Table II and Fig. 5e), as reported in 2D culture on Transwells (19). However, the transport of [^3^H] L-glutamate via GLAST was comparable in the A-to-B and B-to-A directions (Table II and Fig. 5f). In contrast, [^3^H] L-glutamate showed significant transport in the A-to-B direction in 2D cultures, indicating that the transport functions differ between 2D- and 3D-cultured hiPS-BMECs (19). Although EAAT2 (SLC1A2) and EAAT3 (SLC1A1) have been reported to be involved in L-glutamate transport, their expression levels in 2D-cultured hiPS-BMECs were 310-fold and 40-fold lower than that of GLAST, respectively (data not shown), suggesting that GLAST may be predominantly responsible for the transport of [^3^H]L-glutamate in hiPS-BMECs. In rodents and cattle, it has been reported that L-glutamate, a neurotoxin, is excreted from the brain into the blood, and that GLAST expression in astrocytes is mainly responsible for its transport in the BBB (47–49). In the 3D-BBB system, enhanced transport of L-glutamate in the B-to-A direction and additional transport by astrocytes might reproduce the efflux transport from the brain, implying that co-culturing this 3D-BBB system with astrocytes and pericytes could be a promising strategy for future study.

The function of MCT1 (SLC16A1), which is driven by a gradient of H^+^, was assessed by adjusting the pH of the capillary or brain lanes. [^14^C] L-Lactate, a substrate of MCT1, was transported similarly in both directions with both lanes at pH 7.4 (Fig. 5g and Table II). However, when the pH of one of the lanes was adjusted to 6.0, the transport of [^14^C] L-lactate along the H^+^-gradient was enhanced (Fig. 5h, i). This accelerated transport of [^14^C] L-lactate was significantly inhibited by AZD3965, an inhibitor of MCTs (Table II), while pH change might cause a possible alteration in the molecular forms proportion of [^14^C] L-lactate to affect its transport. The rather low expression level of MCT2 in 3D-cultured hiPS-BMECs indicated that MCT1 transports its substrates in both directions in the 3D-BBB system. These results indicate that this 3D-BBB system is sensitive to the H^+^ gradient that might exist between the blood and brain. Metabolic acidosis has been reported to cause alterations in drug transport and transporter expression in the kidney (50, 51), and it is conceivable that evaluation of such changes in the BBB could be performed by using this 3D-BBB system.

The function of LAT1 (SLC7A5) was investigated using gabapentin. LAT1, expressed at the BBB, has been reported to transport drugs such as L-dopa and pregabalin into the brain (52, 53). Our gabapentin transport study showed greater transport in the A-to-B direction than in the B-to-A direction (Table II and Fig. 5j). In addition, it is noteworthy that the *P*_app_ value of gabapentin in the A-to-B direction was decreased by 91% in the presence of 10 µM JPH203, whereas that in the B-to-A direction was decreased by 58% (Table II). These results suggest higher functional expression of LAT1 in the luminal membrane (blood side) than in the abluminal membrane (brain side). These results are also supported by the immunocytochemical finding that the signals of LAT1 and BCRP (a luminal marker protein) were partially merged (Fig. 3), and are consistent with the reported luminal localization of LAT1 in humans and rodents (54). Thus, the 3D-BBB system might also be useful to evaluate efflux transport from the brain side. Since JPH203, an inhibitor of LAT1, decreased gabapentin transport in both directions (Table II), LAT1 may be involved in the influx transport from the blood side and efflux transport from the brain side.

Although hiPS-BMECs show epithelial-like properties (55), they highly expressed VE-cadherin with low expression of CD31. These results suggest that some properties of hiPS-BMECs are not fully developed in comparison to a pure mature endothelial phenotype. Nevertheless, hiPS-BMECs are generally considered to be promising model cells for the BBB, and our results regarding their cell-to-cell tightness and transport function support this view. In order for hiPS-BMECs to more closely mimic the characteristics of pure endothelial cells, a more *in vivo*-like environment and further maturation of hiPS-BMECs themselves may be necessary.

## Conclusion

In the present study, a 3D-BBB system was constructed by culturing hiPS-BMECs in the OrganoPlate^®^ 3-lane device. Expression and functional studies showed the formation of paracellular tightness and the functional expression of many transporters, such as BCRP, MCT1, and LAT1. In addition, the effect of the 3D environment on the expression and localization of several transporters was examined in comparison with a 2D environment. Overall, this 3D-BBB system reproduces many of the characteristics of the human BBB, and is expected to be useful as a high-throughput evaluation tool to facilitate the development of CNS drugs.

## Supplementary Information

Below is the link to the electronic supplementary material.

Supplementary file1 (DOCX 99 kb)
