# Correction to: Construction and Functional Evaluation of a Three-Dimensional Blood–Brain Barrier Model Equipped With Human Induced Pluripotent Stem Cell-Derived Brain Microvascular Endothelial Cells

**DOI:** 10.1007/s11095-022-03264-4

**Published:** 2022-04-20

**Authors:** Toshiki Kurosawa, Daiki Sako, Yuma Tega, Yasuyuki Debori, Yumi Tomihara, Kazunobu Aoyama, Yoshiyuki Kubo, Nobuyuki Amano, Yoshiharu Deguchi

**Affiliations:** 1 Laboratory of Drug Disposition and Pharmacokinetics, Faculty of Pharma-Sciences, Teikyo University, 2-11-1 Kaga, Itabashi, Tokyo, 173-8605 Japan; 2 Axcelead Drug Discovery Partners Inc., 26-1, Muraoka-Higashi 2-chome Fujisawa, Kanagawa, 251-0012 Japan

**Correction to: Pharmaceutical Research**

10.1007/s11095-022-03249-3

This article was updated to correct the phrase “in vitro” in the Abstract and in three instances in the text.

