# Supplementary material for: Construction and Functional Evaluation of a Three-Dimensional Blood–Brain Barrier Model Equipped With Human Induced Pluripotent Stem Cell-Derived Brain Microvascular Endothelial Cells
Source: Pharm Res. 2022 Apr 11;39(7):1535–47. doi: 10.1007/s11095-022-03249-3 (PMC9246774; doi:10.1007/s11095-022-03249-3)
Supplement: Supplementary file 1 — Supplementary file1 (DOCX 99 kb) [file 11095_2022_3249_MOESM1_ESM.docx]

**Supplementary Information**

**Medium contents**

Unconditioned medium: DMEM/F12 (1:1) (Life Technologies, Carlsbad, CA) supplemented with 20% KnockOut^TM^ Serum Replacement (Life Technologies), 0.5% Glutamax (Life Technologies), 1% non-essential amino acids (Life Technologies) and 0.0007% 2-mercaptoethanol (Nacalai tesque, Kyoto, Japan).

**Buffer contents**

Assay buffer in permeability experiment: HBSS buffer (FUJIFILM Wako) containing 10 mM HEPES and 1 mM sodium pyruvate.

**Construction of the 3D-BBB system**


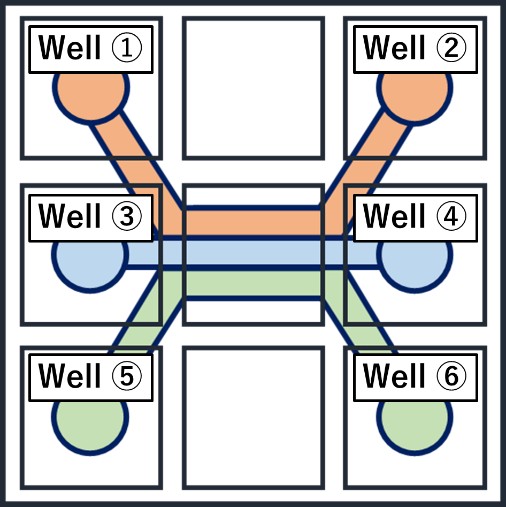
On day 7 during differentiation of hiPS-BMECs, the hydrogel composed of 4 mg/mL collagen I, 100 mM HEPES and 3.7 mg/mL NaHCO_3_ was prepared on ice according to the official protocol, and dispensed into the middle lane. After 15 min incubation, HBSS was added to the gel channel (Well ③ and ④) to prevent the gel from drying out. In addition, the capillary lane was coated with 2 µL of fibronectin/collagen IV. On day 8, after removing the HBSS in Well ③ and ④, hiPS-BMECs was seeded into capillary lane as described in the method section of the main text. In addition, the brain lane was filled with the same medium in the capillary lane. After the plate was tilted and incubation for 4 hr, 50 µL of medium was added to Well ➀ and ②, respectively, and the culture was started on the OrganoFlow^®^. At day 9, the medium in the capillary channels (Well ➀ and ②) was replaced with 50 μL of fresh ESFM + 1% hPDS containing 10 μM MMP inhibitor.

**Permeability experiment in the 3D-BBB system**

On day 10, remove the medium in the capillary lane of the 3D-BBB system. After removal, 100 µL of assay buffer (50 µL in each Well ➀, ②, ⑤ and ⑥) was added to the capillary and brain lane for washing. After 30 min for washing, 100 µL of a fresh assay buffer containing the drug was added to the donor side (A-to-B direction; 50 µL in each Well ➀ and ②, B-to-A direction; 50 µL in each Well ⑤ and ⑥). In addition, 100 µL of a fresh assay buffer was added to the receiver side (A-to-B direction; 50 µL in each Well ⑤ and ⑥, B-to-A direction; 50 µL in each Well ➀ and ②). Inhibitors were added on both sides of the donor and receiver to avoid a gradient in concentration. At the designed sampling time, 50 µL of assay buffer was collected from the receiver side (A-to-B direction; 25 µL in each Well ⑤ and ⑥, B-to-A direction; 25 µL in each Well ➀ and ②), and 50 µL of a fresh assay buffer was added. At 3.5 hr after the start of permeability experiment, the transport amount of lucifer yellow on the receiver side was measured to check for leakage, and all donor and receiver were collected at 4 hr. The donor sample was measured by LC-MS/MS as well as the receiver sample to confirm that the drug was not degraded.

**LC-MS/MS analysis**

Samples were diluted with methanol and the mobile phase containing the internal standard alprenolol. The mobile phase for gradient analysis was composed of solvent A (10 mM ammonium formate buffer containing 0.2% formate) and solvent B (methanol). The gradient was programmed as follows: 0-1 min 0% B, 1-2 min linear increase to 95% B, kept constant to 4 min, and finally the initial condition was restored and held for 1 min to re-equilibrate the system. Chromatographic separation was achieved in a Synergi Hydro-RP column (2.0 × 50 mm, 2.5 µm, Phenomenex, Torrance, CA) at a flow rate of 0.4 mL/min. The column temperature was set at 40^o^C. Analyst 1.6.1 software was used to collect data and to control the QTRAP4500 system. The MRM transitions of test compounds are shown in Supporting Table 1.

**Supporting Table 1. MRM transitions of test compounds.**

| Test compounds | MRM transition | |
| --- | --- | --- |
|  | Q1 Mass | Q3 Mass |
| Alprenolol | 250.2 | 116.0 |
| Antipyrine | 188.9 | 77.0 |
| Atenolol | 267.2 | 144.9 |
| Cladribine | 286.2 | 170.0 |
| Dantrolene | 315.1 | 114.0 |
| Gabapentin | 171.9 | 154.0 |
| Quinidine | 325.1 | 307.1 |

**Supporting Table 2. Sequences of sense and antisense primers used for qPCR.**

| **Target mRNA** | **Sequence (5’-3’)** | **Tm**  **(^o^C)** | **Product size**  **(bp)** | **Ct value** | |
| --- | --- | --- | --- | --- | --- |
|  |  |  |  | **2D culture** | **3D cuture** |
| ABCB1 (P-gp) | CCATGCTCAGACAGGATGTG | 64.1 | 121 | 41.5 | 41.1 |
|  | ACAGCAAGCCTGGAACCTAT | 62.2 |  |  |  |
| ABCC1 (MRP1) | GCCGAAGGAGAGATCATC | 59.4 | 109 | - | - |
|  | AACCCGAAAACAAAACAGG | 60.9 |  |  |  |
| ABCC4 (MRP4) | GCTCACGCGTGTTCTTCTGG | 67.9 | 138 | 38.7 | 30.9 |
|  | CCCAGAACCCTTGCAACTCCT | 68.1 |  |  |  |
| ABCC5 (MRP5) | ATCGCCTGCACACGGTTCTA | 67.5 | 133 | 34.3 | 31.7 |
|  | TCTGCAGCAGCAAACATGGC | 69.5 |  |  |  |
| ABCG2 (BCRP) | GGCTTTGCAGCATAATGAATTTTT | 65.3 | 71 | 31.7 | 28.7 |
|  | AAGGATTGTTTCCTGTTGCATTG | 65.5 |  |  |  |
| SLC1A3 (GLAST) | CGACATCACGCTCATCATCGC | 70.9 | 72 | 29.0 | 28.2 |
|  | TCTCCCAGTACGTTGGTGGTG | 66.7 |  |  |  |
| SLC2A1 (GLUT1) | CTGAGCTCCTCCAGTCTGATGA | 67.1 | 127 | 32.1 | 28.1 |
|  | CTCTGATTCCGGCTCCTTCTC | 67.7 |  |  |  |
| SLC2A3 (GLUT3) | GGTGCTGCTCACGTCTCTCT | 64.4 | 147 | 31.8 | 31.0 |
|  | CCACCAGTGACAGCCAACAG | 66.5 |  |  |  |
| SLC3A2 (4F2hc) | CCGGCTCAACTTCTCCGACT | 67.4 | 125 | 32.6 | 28.5 |
|  | GGAGCCTCCATAGGCTGTCC | 66.7 |  |  |  |
| SLC5A7 (CHT1) | TGTTGTTGATGCTGGGTGGAATC | 69.6 | 75 | 32.7 | 32.0 |
|  | CATAGGTGGCTGAGGAAGAAGAG | 64.7 |  |  |  |
| SLC7A1 (CAT1) | GCTCACACACACCCTTGTCCAA | 65.8 | 75 | 34.2 | 30.9 |
|  | AATGCATCTATCACTGTCCTCTTGAT | 64.4 |  |  |  |
| SLC7A5 (LAT1) | GGAACATTGTGCTGGCATTATACA | 66.1 | 75 | 28.6 | 27.2 |
|  | CCTCTGTGACGAAATTCAAGTAATTC | 64.3 |  |  |  |
| SLC7A6 (LAT2) | AGCCCTGAAGAAAGAGATCG | 62.0 | 530 | - | - |
|  | TGCATATCTGTACAATCCCC | 59.4 |  |  |  |
| SLC16A1 (MCT1) | TGAAACATTGATGGACCTTGTTG | 65.3 | 70 | 30.4 | 28.8 |
|  | TTCCACAATGGTCACCAATCC | 66.3 |  |  |  |
| SLC16A7 (MCT2) | CCAAAGCTGTCACCGTATTCTTC | 65.3 | 145 |  | 34.6 |
|  | CTGCCGTATTTATTCACCAAAACAC | 65.6 |  |  |  |
| SLC19A1 (RFC1) | GGGAGCAGGTCACGAACGAG | 69.1 | 97 | 32.1 | 31.7 |
|  | GTGTAGCGCAGGTAGTCGGT | 63.8 |  |  |  |
| SLCO1A2 (OATP1A2) | TATGATTCCACCACCTTCAGATACA | 64.8 | 92 | **-** | - |
|  | AGAATTAAGATGATTAAGGCTGGAACA | 64.3 |  |  |  |
| SLCO1B1 (OATP1B1) | CAACAGTATGGTCAGCCTTCATCT | 64.9 | 76 | - | - |
|  | TTCCACTTGCAAAAATAGGTATGG | 64.2 |  |  |  |
| SLCO2A1 (OATP2A1) | GCCAGCACTGGGAACAACAG | 67.7 | 81 | 33.6 | 34.6 |
|  | GTGGCACTTACTGGGAGGCA | 67.0 |  |  |  |
| SLC22A1 (OCT1) | AATGGACCACATCGCTCAAAA | 66.0 | 68 | 33.9 | 35.2 |
|  | CTTCGAGGGAAAGCATCTTTAAAT | 63.9 |  |  |  |
| SLC22A2 (OCT2) | GGACGGCTGGGTGTACGA | 66.9 | 70 | - | - |
|  | GGAGTTGGCACATACCAGGTTAA | 65.4 |  |  |  |
| SLC22A3 (OCT3) | CATGCCTTGTCACTGCGTTCT | 67.1 | 63 | 34.2 | 36.3 |
|  | ATGTAGCCACTGTGGTCCTCAA | 65.2 |  |  |  |
| SLC22A4 (OCTN1) | CCAGAGTAGGCAGCATCAT | 66.0 | 67 | 38.3 | 40.8 |
|  | TAGGGCAGCATTCTGTTGTAAGC | 65.8 |  |  |  |
| SLC22A5 (OCTN2) | CCTTCTCTTCATGCAGCTGGTA | 65.1 | 66 | 31.9 | 32.9 |
|  | CCCACCATCACCAGGACTGT | 66.9 |  |  |  |
| SLC22A6 (OAT1) | GGCTGGCATCTCCCTCAAC | 66.7 | 65 | - | - |
|  | AGGCCCGTGTGTGAATGG | 66.8 |  |  |  |
| SLC22A7 (OAT2) | CCATCACCCGCACCCTTAC | 67.1 | 65 | 35.8 | 33.4 |
|  | CTCCAGTGGCATCACGATGA | 67.1 |  |  |  |
| SLC22A8 (OAT3) | CGCTTCCTGTGTGGCTTTG | 66.5 | 68 | - | - |
|  | ACCCATTCCACATTCAAGATGAC | 65.5 |  |  |  |
| SLC29A1 (ENT1) | ACGGCCACTCAGTATTTCACAA | 65.1 | 81 | 31.5 | 30.6 |
|  | GGCGTCCTTGCTCAGTTCAG | 66.9 |  |  |  |
| SLC29A2 (ENT2) | CTGAGCTCCTCCAGTCTGATGA | 65.5 | 98 | 33.8 | 32.2 |
|  | CTCTGATTCCGGCTCCTTCTC | 65.9 |  |  |  |
| SLC29A4 (PMAT) | CAGCTTTCACGGATACTACATTGG | 65.0 | 67 | 33.1 | 32.3 |
|  | GCAAAGTAGATGGCGTGATAACG | 66.0 |  |  |  |
| SLC35F2 | ATTGCTTTGGGATTCCTGTG | 63.3 | 114 | 34.6 | 33.1 |
|  | CCATGGTTCCTACACCCAAC | 63.6 |  |  |  |
| SLC43A3 (ENBT1) | CTCAGCCGTGCTGCTCTTC | 66.1 | 78 | 34.8 | 34.1 |
|  | ATCTGCAGGTTGGTGATGAGAA | 65.4 |  |  |  |
| SLC44A1 (CTL1) | GCGTGTCCAAGGCAAGAACT | 66.1 | 69 | 30.9 | 30.3 |
|  | GGCTGAACCATTTATCTCTGCAA | 65.7 |  |  |  |
| SLC44A2 (CTL2) | CTCATCGCGATTGCACTCA | 66.0 | 66 | 29.7 | 29.3 |
|  | GAGCAAGGAGCACATGACGTATC | 66.5 |  |  |  |
| SLC47A1 (MATE1) | CAGTCACGCTGGCAATCG | 66.6 | 77 | - | - |
|  | GGTGTCACAGGCAGAAGATAAGC | 65.6 |  |  |  |
| SLC47A2 (MATE2) | TCTGAGAGGAACTGGGAAGCA | 65.9 | 69 | 33.2 | 33.1 |
|  | AGGCCGATGATGTAATATGTGATG | 65.1 |  |  |  |
| OSCP1 | CCAGTCCAGCTCGTTTCCA | 66.1 | 68 | 33.6 | 35.0 |
|  | CCGCCCAAGTTCAAGAAGAG | 65.5 |  |  |  |
| LEPR | GGAAATCACACGAAATTCAC | 59.0 | 139 | - | - |
|  | GCACGATATTTACTTTGCTC | 56.1 |  |  |  |
| LRP1 | GAGGGAGTGACCCACGTCTG | 66.8 | 87 | 32.1 | 32.0 |
|  | AATCACACACCCACGCTTTGC | 68.0 |  |  |  |
| TFRC | CGGCTGCAGGTTCTTCTGTG | 67.9 | 143 | - | - |
|  | CTCCACATGACTGTTATCGCCATC | 68.1 |  |  |  |
| RAGE | ATTGGTGGTGGAGCCAGAAG | 65.9 | 114 | 34.3 | 32.5 |
|  | GGCACACCATCCTTCATCCA | 67.9 |  |  |  |
| INSR | GTGTGGCAGCCTACGTCAGT | 64.9 | 136 | 33.9 | 31.6 |
|  | AGACCATTGGGCTCCTTCGG | 69.1 |  |  |  |
| GLP1R | TCCTTCATCCTGCGAGCATT | 66.6 | 74 | 30.9 | 31.4 |
|  | TGGGCGGCTGTGCTATACA | 67.2 |  |  |  |
| GAPDH | CCACATCGCTCAGACACCAT | 65.9 | 66 | 25.6 | 26.0 |
|  | GCGCCCAATACGACCAAAT | 66.4 |  |  |  |

Sequences of primers were based on previous reports (Shimomura K *et al*, Fluids Barriers CNS, 20(1):8, 2013 and Yamamoto A *et al*, J Pharm Sci, 99(5):2475-82, 2010, etc.), but specificity of primers was not verified.

**Supporting Table 3. Product information used to culture hiPS-BMECs and construct 2D and 3D cultures.**

| **Product Name** | **Manufacturer** | **Product Number** |
| --- | --- | --- |
| **Culture of hiPS-BMECs** | | |
| mTeSR1-cGMP | StemCell Technologies | 85851 |
| Matrigel^®^ Basement Membrane Matrix Growth Factor Reduced | Corning | 356230 |
| 6-well plate | Thermo Fisher Scientific | 140675 |
| Accutase | Merck-Millipore | SF006 |
| Y27632 (ROCK inhibitor) | FUJIFILM Wako | 259-00613 |
| DMEM/F12 (1:1) | Life Technologies | 11330-032 |
| KnockOut^TM^ Serum Replacement | Life Technologies | 10828-028 |
| Glutamax | Life Technologies | 35050-061 |
| non-essential amino acids | Life Technologies | 11140-050 |
| 2-mercaptoethanol | Nacalai tesque | 21438-82 |
| human endothelial serum-free medium | Thermo Fisher Scientific | 11111-04 |
| Human Serum from platelet poor human plasma (hPDS) | Sigma-Aldrich Company | P2918-100ML |
| all-trans-retinoic acid | FUJIFILM Wako | 186-01114 |
| human fibroblast growth factor 2 | Sigma-Aldrich Company | F0291-25UG |
| **Construct 2D and 3D cultures** | | |
| HBSS (+) without phenol red | FUJIFILM Wako | 084-08965 |
| fibronectin/collagen IV | PharmaCo-Cell | PCC-BECCS-10 |
| Cell culture insert (Transparent PET Membrane, 24 well, 0.4 µm pore size) | CORNING | 353095 |
| 24-well plate for use with cell culture insert | CORNING | 353504 |
| OrganoPlate^®^ 3-lane plates | MIMETAS | 4004-400-B |
| OrganoFlow^®^ | MIMETAS | MI-OFPR-L |
| Cultrex 3D Matrix Rat Collagen-I | AMSbio | 3447-020-01 |
| GM6001, MMP inhibitor | Abcam | ab120845 |
| **Staining of hiPS-BMECs** | | |
| anti-VE-cadherin (D87F2) | Cell Signaling Technology | 2500S |
| anti-GLUT1 (SPM498) | Abcam | ab40084 |
| anti-claudin-5 (4C3C2) | Thermo Fisher Scientific | 35-2500 |
| anti-ZO-1 | Thermo Fisher Scientific | 40-2200 |
| anti-BCRP (5D3) | Merck-Millipore | MAB4155 |
| anti-LAT1 | ATLAS ANTIBODIES | HPA052673 |
| CellMask^TM^ Green plasma membrane stain | Thermo Fisher Scientific | C37608 |
